# Association Between Systemic Anticoagulation and Outcomes in Patients With Acute Respiratory Distress Syndrome Receiving Venovenous Extracorporeal Membrane Oxygenation: Insights From a Multicenter Propensity-Weighted Study

**DOI:** 10.1097/CCM.0000000000007071

**Published:** 2026-02-19

**Authors:** Ginga Suzuki, Kohei Ishikawa, Saria Nishioka, Toshimitsu Kobori, Yuka Masuyama, Saki Yamamoto, Hibiki Serizawa, Yoshimi Nakamichi, Mitsuru Honda, Japan Chest CT for acute respiratory distress syndrome requiring venovenous extracorporeal membrane oxygenation (J-CARVE) Registry group, Mitsuaki Nishikimi

**Affiliations:** Department of Emergency and Critical Care Medicine, Graduate School of Biomedical and Health Sciences, Hiroshima University, Hiroshima, Japan; Department of Emergency and Critical Care Medicine, Graduate School of Biomedical and Health Sciences, Hiroshima University, Hiroshima, Japan; Department of Critical Care and Emergency Medicine, Tokyo Metropolitan Tama Medical Center, Tokyo, Japan; Department of Critical Care and Emergency Medicine, Tokyo Metropolitan Tama Medical Center, Tokyo, Japan; Advanced Medical Emergency Department and Critical Care Center, Japan Red Cross Maebashi Hospital, Maebashi, Japan; Department of Emergency Medicine and Critical Care Medicine, SAISEIKAI Utsunomiya Hospital, Utsunomiya, Japan; Department of Emergency Medicine, Sapporo Medical University, Sapporo, Japan; Department of Emergency Medicine, Sapporo Medical University, Sapporo, Japan; Department of Emergency medicine and Critical care, Fukuoka University Hospital, Fukuoka, Japan; Department of Emergency Medicine and Critical Care Medicine, SAISEIKAI Utsunomiya Hospital, Utsunomiya, Japan; Advanced Medical Emergency Department and Critical Care Center, Japan Red Cross Maebashi Hospital, Maebashi, Japan; Department of Critical Care and Emergency Medicine, Tokyo Metropolitan Tama Medical Center, Tokyo, Japan; Advanced Medical Emergency Department and Critical Care Center, Japan Red Cross Maebashi Hospital, Maebashi, Japan; Department of Emergency and Critical Care Medicine, Graduate School of Biomedical and Health Sciences, Hiroshima University, Hiroshima, Japan; Department of Emergency and Critical Care Medicine, Graduate School of Biomedical and Health Sciences, Hiroshima University, Hiroshima, Japan; 1 All authors: Critical Care Center, Toho University Omori Medical Center, Tokyo, Japan.

**Keywords:** critical care, intensive care, respiratory failure

## Abstract

**OBJECTIVE:**

To evaluate whether systemic anticoagulation therapy affects the survival of adult patients receiving venovenous extracorporeal membrane oxygenation (VV-ECMO) for acute respiratory distress syndrome (ARDS).

**DESIGN:**

Multicenter retrospective study.

**SETTING:**

Twenty-four ICUs in Japan.

**PATIENTS:**

Six hundred and ninety-five patients received VV-ECMO for ARDS. Patients were divided into the anticoagulation group and the no-anticoagulation group according to whether or not they received anticoagulant therapy.

**INTERVENTIONS:**

None.

**MEASUREMENTS AND MAIN RESULTS:**

In the propensity score-overlap-weighted analysis, there was no significant difference in the 28-day survival (85.8% vs. 81.5%, *p* = 0.50) between the two groups. The 60-day survival, ECMO duration, circuit exchanges, bleeding complications, and transfusion volumes were also comparable. The anticoagulation group had a significantly higher average activated partial thromboplastin time during ECMO (51.3 s vs. 39.3 s, *p* < 0.01). These findings remained consistent in the sensitivity analysis using inverse probability of treatment weighting.

**CONCLUSIONS:**

Systemic anticoagulation was not associated with short-term survival. Anticoagulation-free VV-ECMO may be feasible in patients at high-bleeding risk, but safety remains uncertain. Further studies should clarify optimal anticoagulation strategies.

**KEY POINTS**

**Question:** The aim of this study was to evaluate whether systemic anticoagulation therapy affects the survival of adult patients receiving venovenous extracorporeal membrane oxygenation (VV-ECMO) for acute respiratory distress syndrome (ARDS).

**Findings:** In the propensity score (PS)-overlap-weighted analysis, anticoagulant therapy was not associated with differences in outcomes. The 28-day survival (85.8% vs. 81.5%, *p* = 0.50) did not differ significantly between the two groups, and the 60-day survival, ECMO duration, circuit exchanges, bleeding complications, and transfusion volumes were also comparable.

**Meanings:** Systemic anticoagulation may not be necessary in patients with ARDS undergoing VV-ECMO.

## BACKGROUND

Venovenous extracorporeal membrane oxygenation (VV-ECMO) is a lifesaving therapy for severe acute respiratory distress syndrome (ARDS) when conventional ventilation fails (1–15). Although its use has increased worldwide (1), the optimal anticoagulation strategy during VV-ECMO remains uncertain (3–9). Systemic anticoagulation is routinely administered to prevent circuit thrombosis, yet it substantially increases the risk of bleeding—a major contributor to morbidity and mortality in ECMO patients (2, 3).

Emerging reports suggest that anticoagulation-free VV-ECMO may be feasible in selected patients with high-bleeding risk (10–13). Single-center experiences and pediatric data support the possibility of managing ECMO with minimal or no anticoagulation (14). However, robust evidence in adult VV-ECMO populations is limited, and prior studies have primarily compared anticoagulation intensity (e.g., low vs. standard activated partial thromboplastin time [APTT]) rather than evaluating the complete avoidance of anticoagulation (6, 9). Thus, the clinical effect of systemic anticoagulation on outcomes during VV-ECMO remains unclear.

We hypothesized that routine systemic anticoagulation may not be necessary for all VV-ECMO patients. If contemporary ECMO circuits and management strategies reduce thrombotic complications sufficiently, avoiding anticoagulation could lower bleeding and transfusion burden without compromising survival.

Using data from the multicenter Japan Chest CT for ARDS requiring VV-ECMO (J-CARVE) Registry (15), we evaluated whether systemic anticoagulation therapy affects 28-day survival in adult patients receiving VV-ECMO for ARDS.

## METHODS

### Design and Setting

This retrospective study was conducted at Toho University Omori Medical Center, a tertiary emergency medical facility in Tokyo, Japan. The study protocol was approved by the Ethics Committee of Toho University School of Medicine (approval number: T2025-003; approval date: May 12, 2025; study title: “Association Between Anticoagulation Use During Extracorporeal Membrane Oxygenation and Clinical Outcomes”). This study was carried out in compliance with the ethical principles of the Declaration of Helsinki (1975). The need for written informed consent was waived owing to the nature of the study. Information about the study was disclosed on the institutional website, providing potential participants with the opportunity to opt-out.

### Participants

All the patients enrolled in the J-CARVE Registry were included in this study. The J-CARVE is a multicenter retrospective registry in which data on patients receiving VV-ECMO for ARDS in Japan are captured (15). The registry includes data from 24 participating centers covering a wide range of VV-ECMO practices and patient populations. It contains detailed information on patient characteristics, VV-ECMO management, anticoagulation strategies, and clinical outcomes.

### Exclusion Criteria

Patients with missing data on anticoagulant use were excluded.

### Data Extraction

Data from the J-CARVE Registry were collected between January 1, 2012 and December 31, 2022. We included patients treated from November 7, 2012 onward, when complete VV-ECMO and anticoagulation data became available. The registry contains baseline demographics, comorbidities, ARDS etiology (pulmonary vs. non-pulmonary), pre-ECMO laboratory results, gas exchange variables (including P/F ratio), the sequential organ failure assessment (SOFA) score (16), and the interval from mechanical ventilation to VV-ECMO initiation.

Pre-ECMO ventilator were also recorded. Detailed membrane oxygenator readouts—including pre/post-membrane pressures and ECMO parameters at decannulation—were not available.

Anticoagulation status was captured as a binary variable (yes/no). In Japan, unfractionated heparin is the standard anticoagulant; however, its use in clinical settings are not standardized and are made at the discretion of clinicians at each institution. Although explicit reasons for withholding anticoagulation were not recorded, real-world no-anticoagulation VV-ECMO is chosen for patients with substantial bleeding risk.

### Outcome

The primary outcome was 28-day survival, selected as a clinically relevant measure of early mortality in patients receiving VV-ECMO and the period during which the effects of anticoagulation—both benefit and harm—would be expected to appear.

Secondary outcomes included 60-day survival, ECMO duration, circuit exchange frequency, bleeding complications, mean APTT during VV-ECMO, and transfusion requirements (red cell concentrate [RCC], fresh frozen plasma [FFP], and platelet concentrate [PLT]).

### Statistical Analysis

Continuous variables are expressed as medians with interquartile ranges and were compared using the Mann–Whitney *U* test. Categorical variables are expressed as percentages and were compared using the chi-square test or Fisher exact test. For multiple comparisons across outcomes, the Benjamini–Hochberg (BH) correction (17, 18) was applied to adjust *p* values accordingly.

To evaluate the impact of anticoagulation use, we analyzed the mean APTT levels from days 1 to 7 during VV-ECMO support. For each day, two-group comparisons were conducted using the Mann–Whitney *U* test, and the BH correction was applied to adjust for multiple comparisons. APTT trends were visualized using a line plot with se bars and statistically significant differences were marked accordingly.

To account for confounding factors, overlap weighting based on propensity score (PS) (19, 20) was used as the primary analysis. The PS was estimated using a logistic regression model incorporating age, sex, height, weight, blood tumor, cirrhosis, ARDS etiology (pulmonary or non-pulmonary origin), APTT, hemoglobin level, platelet count, fibrinogen level, and SOFA score. These variables were selected based on their potential influence on anticoagulation therapy. Overlap weighting was chosen for its ability to approximate a randomized controlled trial by balancing the covariates between treatment groups. Weights were calculated as p(1–p), where p represents the estimated PS (19).

To avoid extreme weights that could introduce instability, the weights were trimmed to the first and 99th percentiles (21, 22). The balance of covariates before and after weighting was assessed using standardized mean differences (SMD), with SMD less than 0.1 considered well-balanced (23), and SMD less than 0.25 considered tolerable (24). Because anticoagulation practices vary among institutions in Japan, several baseline laboratory variables were interpreted as proxy markers reflecting site-level anticoagulation tendencies and bleeding-risk management. These variables were therefore incorporated into the PS model to partially adjust for heterogeneity in institutional practice patterns.

The primary outcome was 28-day survival, whereas secondary outcomes included 60-day survival, total complications, circuit exchange frequency, and transfusion requirements (RCC, FFP, and PLT). After weighting, univariate regression analyses were performed to assess the association between anticoagulation therapy and each outcome. Overlap-weighted logistic regression was used for binary outcomes and overlap-weighted simple regression was used for continuous outcomes.

For the sensitivity analysis, inverse probability of treatment weighting (IPTW) (25, 26) was performed using stabilized weights to confirm the robustness of our findings. Similar to the primary analysis, the balance before and after weighting was assessed using the SMD, and univariate regression analyses were conducted for each outcome.

In addition, we conducted three supplementary sensitivity analyses using the same PS estimated in the main analysis, with 28-day survival as the dependent variable in all the models. First, we performed multivariable logistic regression including the PS, anticoagulation use, platelet count, and fibrinogen level as covariates. Second, we constructed another multivariable logistic regression model with the PS, anticoagulation use, and ARDS origin (pulmonary vs. non-pulmonary) as explanatory variables. Third, we conducted Bayesian logistic regression using the PS and anticoagulant use as covariates. The Bayesian model was fitted using the rstanarm package with weakly informative priors (Normal [0, 2.5] for the regression coefficients). Four Markov Chain Monte Carlo chains were run for 2000 iterations each, with 1000 warm-up iterations. All chains converged successfully (all *R̂* < 1.01), and the effective sample sizes were adequate to support robust inference.

We further assessed potential institutional heterogeneity in anticoagulation practice by fitting a mixed-effects Cox proportional hazards model with a facility-level random intercept, applying the same overlap weights used in the primary analysis. Furthermore, to examine whether ECMO management factors influenced treatment decisions or outcomes, we repeated the overlap-weighted logistic regression including pump flow rate at ECMO initiation as an additional covariate. Finally, to account for potential temporal changes in clinical practice (e.g., due to COVID-19), we derived the calendar year of each hospitalization and incorporated it into an overlap-weighted logistic regression model using the same PS.

To further illuminate factors that led to a withholding of anticoagulation, we visualized daily transfusion volumes and the cumulative incidence of bleeding complications.

All statistical analyses were performed using R, Version 4.5.0 (R Foundation for Statistical Computing, Vienna, Austria), with statistical significance set at *p* value of less than 0.05.

## RESULTS

Key baseline characteristics are shown in **Table 1**, and the full dataset is provided in **eTable 1** (https://links.lww.com/CCM/H903). The study included 641 and 54 patients in the anticoagulation and no-anticoagulation groups, respectively. There were no significant differences between the two groups in terms of age, sex, height, weight, or SOFA score.

**TABLE 1.** Baseline Patient Characteristics

Parameters	Anticoagulation Group (*n* = 641)	No-Anticoagulation Group (*n* = 54)	*p*
Age, yr	60.0 (50.0–68.0)	58.5 (47.3–66.0)	0.17
Male, *n* (%)	489 (76.3)	44 (81.5)	0.48
Height, cm	167.0 (160.0–172.0)	165.0 (160.0–170.0)	0.40
Weight, kg	72.2 (61.0–85.1)	69.0 (58.0–80.0)	0.17
Medical history			
Diabetes, *n* (%)	205 (32.0)	9 (16.7)	0.03
Chronic obstructive pulmonary disease, *n* (%)	43 (6.7)	0 (0)	0.04
Asthma, *n* (%)	46 (7.2)	2 (3.7)	0.57
Interstitial lung disease, *n* (%)	26 (4.1)	1 (1.9)	0.71
Blood tumor, *n* (%)	14 (2.2)	2 (3.7)	0.36
Cirrhosis, *n* (%)	11 (1.7)	1 (1.9)	1.00
Pulmonary-origin acute respiratory distress syndrome, *n* (%)	605 (94.4)	44 (81.5)	< 0.01
Laboratory data before ECMO			
pH	7.3 (7.2–7.4)	7.3 (7.2–7.4)	0.46
Lactate, mmol/L	1.7 (1.2–2.4)	2.0 (1.3–4.4)	0.02
Hemoglobin, g/dL	12.5 (10.5–14.1)	11.0 (9.7–13.0)	< 0.01
Platelet count, ×10^3^/μL	184.0 (115.0–257.0)	116.0 (75.0–163.0)	< 0.01
C-reactive protein, mg/dL	12.6 (4.9–21.7)	8.2 (2.1–16.5)	0.05
Albumin, mg/dL	2.5 (2.0–2.8)	2.5 (2.3–3.0)	0.07
Creatinine, mg/dL	0.9 (0.7–1.5)	1.0 (0.7–1.9)	0.43
Activated partial thromboplastin time, s	35.0 (29.0–46.5)	32.0 (28.9–37.3)	0.09
Fibrinogen/fibrin degradation products, μg/mL (*n* = 398, 36)	14.1 (6.6–36.1)	24.4 (8.9–50.2)	0.30
Fibrinogen, mg/dL (*n* = 588, 49)	509.0 (347.8–657.5)	365.0 (254.0–614.0)	0.01
Sequential organ failure assessment score	10.0 (7.0–12.0)	10.5 (8.0–13.0)	0.24
Duration between MV and ECMO initiation, d	2.0 (1.0–5.0)	2.0 (1.0–5.0)	0.59
Gas exchange status before ECMO			
Fio_2_	1.0 (0.8–1.0)	1.0 (1.0–1.0)	0.04
Po_2_, torr	71.4 (60.0–86.1)	72.0 (56.8–83.0)	0.24
P/F ratio	81.5 (64.0–104.1)	81.4 (62.0–97.0)	0.44
Pco_2_, torr	49.0 (41.0–62.7)	47.0 (37.1–62.7)	0.52
MV setting before ECMO			
Positive end-expiratory pressure, cm H_2_O	12.0 (10.0–15.0)	10.0 (8.0–15.0)	0.02
Tidal volume, mL (*n* = 496, 46)	400.0 (322.5–477.3)	380.0 (301.5–450.0)	0.21

ECMO = extracorporeal membrane oxygenation, MV = mechanical ventilation.

Data are presented as median (interquartile range) or *n* (%). Unadjusted comparisons were performed using the Mann-Whitney *U* test for continuous variables and the chi-square or Fisher exact test for categorical variables.

However, several differences were observed between the groups. The proportion of patients with pulmonary-origin ARDS was significantly higher in the anticoagulation group (94.4% vs. 81.5%, *p* < 0.01). In addition, the anticoagulation group had a higher prevalence of hypertension (39.6% vs. 24.1%, *p* = 0.03), diabetes (32.0% vs. 16.7%, *p* = 0.03), and chronic obstructive pulmonary disease (6.7% vs. 0%, *p* = 0.04).

Regarding laboratory data before VV-ECMO initiation, the anticoagulation group had significantly higher hemoglobin levels (12.5 vs. 11.0 g/dL, *p* < 0.01), platelet counts (184.0 vs. 116.0 × 10^3^/μL, *p* < 0.01), and fibrinogen levels (509.0 vs. 365.0 mg/dL, *p* = 0.01). In contrast, the total bilirubin level was significantly higher in the no-anticoagulation group (*p* = 0.02), while lactate levels were lower in the anticoagulation group (1.7 vs. 2.0 mmol/L, *p* = 0.02). Other laboratory parameters, including pH and creatinine levels were comparable between the groups. With respect to gas exchange before VV-ECMO initiation, Fio_2_ on the ventilator was significantly lower in the anticoagulation group (*p* = 0.04), whereas pH, Po_2_, Pco_2_, and the calculated P/F ratio were similar between the groups.

In terms of mechanical ventilation settings before VV-ECMO, positive end-expiratory pressure was higher in the anticoagulation group (*p* = 0.02), whereas tidal volume was similar between the two groups (*p* = 0.21).

At ECMO initiation, ECMO Fio_2_ was uniformly set at 1.0 in both groups (*p* = 0.24). The pump flow rate was slightly higher in the no-anticoagulation group (4.1 [3.5–4.7] vs. 4.0 [3.5–4.2] L/min), and the difference reached statistical significance (*p* = 0.01). Rotation speed (*p* = 0.20) and sweep gas flow rate (*p* = 0.67) did not differ significantly between the groups (**eTable 2**, https://links.lww.com/CCM/H903).

The 28-day survival rates were similar between the two groups (85.8% vs. 81.5%, *p* = 0.50). The 60-day survival rate showed no significant difference after adjustment (74.7 vs. 66.7%, unadjusted *p* = 0.19, adjusted *p* = 0.23). The ECMO duration tended to be longer in the anticoagulation group (11.0 [7.0–19.0] vs. 9.0 [6.0–16.8] days, unadjusted *p* = 0.05, adjusted *p* = 0.09). The number of ECMO circuit exchanges and incidence of bleeding complications did not differ significantly between the groups (unadjusted *p* = 0.17 and *p* = 0.21, and adjusted *p* = 0.23 for both) (**Table 2**).

**TABLE 2.** Patient Outcomes

Parameters	Anticoagulation Group (*n* = 641)	No-Anticoagulation Group (*n* = 54)	Unadjusted *p*	Adjusted *p*
28-d survival, *n* (%)	550 (85.8)	44 (81.5)	0.50	
60-d survival, *n* (%)	479 (74.7)	36 (66.7)	0.19	0.23
ECMO duration, d	11.0 (7.0–19.0)	9.0 (6.0–16.8)	0.05	0.09
Number of ECMO circuit exchanges, *n*	0.0 (0.0–1.0)	0.0 (0.0–1.0)	0.17	0.23
Bleeding complication, *n* (%)	201 (31.4)	22 (40.7)	0.21	0.23
Average activated partial thromboplastin time, s	51.3 (44.8–59.2)	39.3 (35.4–46.2)	<0.01	< 0.01
Transfusion volume				
Red cells concentrate, units	6.0 (0.0–14.0)	13.0 (6.0–26.5)	<0.01	< 0.01
Fresh frozen plasma, units	0.0 (0.0–8.0)	7.0 (0.0–43.5)	<0.01	< 0.01
Platelet concentrate, units	0.0 (0.0–20.0)	15.0 (0.0–65.0)	< 0.01	< 0.01

ECMO = extracorporeal membrane oxygenation.

Data are presented as median (interquartile range) or *n* (%). Unadjusted comparisons were performed using the Mann-Whitney *U* test for continuous variables and the chi-square or Fisher exact test for categorical variables. Adjusted *p* values were derived from overlap-weighted regression models. Average activated partial thromboplastin time calculated for each patient across venovenous-ECMO days 1–7.

However, the average APTT during VV-ECMO support was higher in the anticoagulation group (51.3 [44.8–59.2] vs. 39.3 [35.4–46.2] s, *p* value of less than 0.01 for both unadjusted and adjusted comparisons). In contrast, the transfusion volumes were significantly higher in the no-anticoagulation group. Patients without anticoagulation required more RCC (13.0 [6.0–26.5] vs. 6.0 [0.0–14.0] units, *p* < 0.01), FFP (7.0 [0.0–43.5] vs. 0.0 [0.0–8.0] units, *p* < 0.01), and platelet transfusions (15.0 [0.0–65.0] vs. 0.0 [0.0–20.0] units, *p* < 0.01), even after adjustment (Table 2).

A comparison of APTT between the two groups extracted from VV-ECMO days 1–7 revealed significant differences between days 2 and 7 (*p* < 0.05; **Fig. 1**).

**Figure 1.**
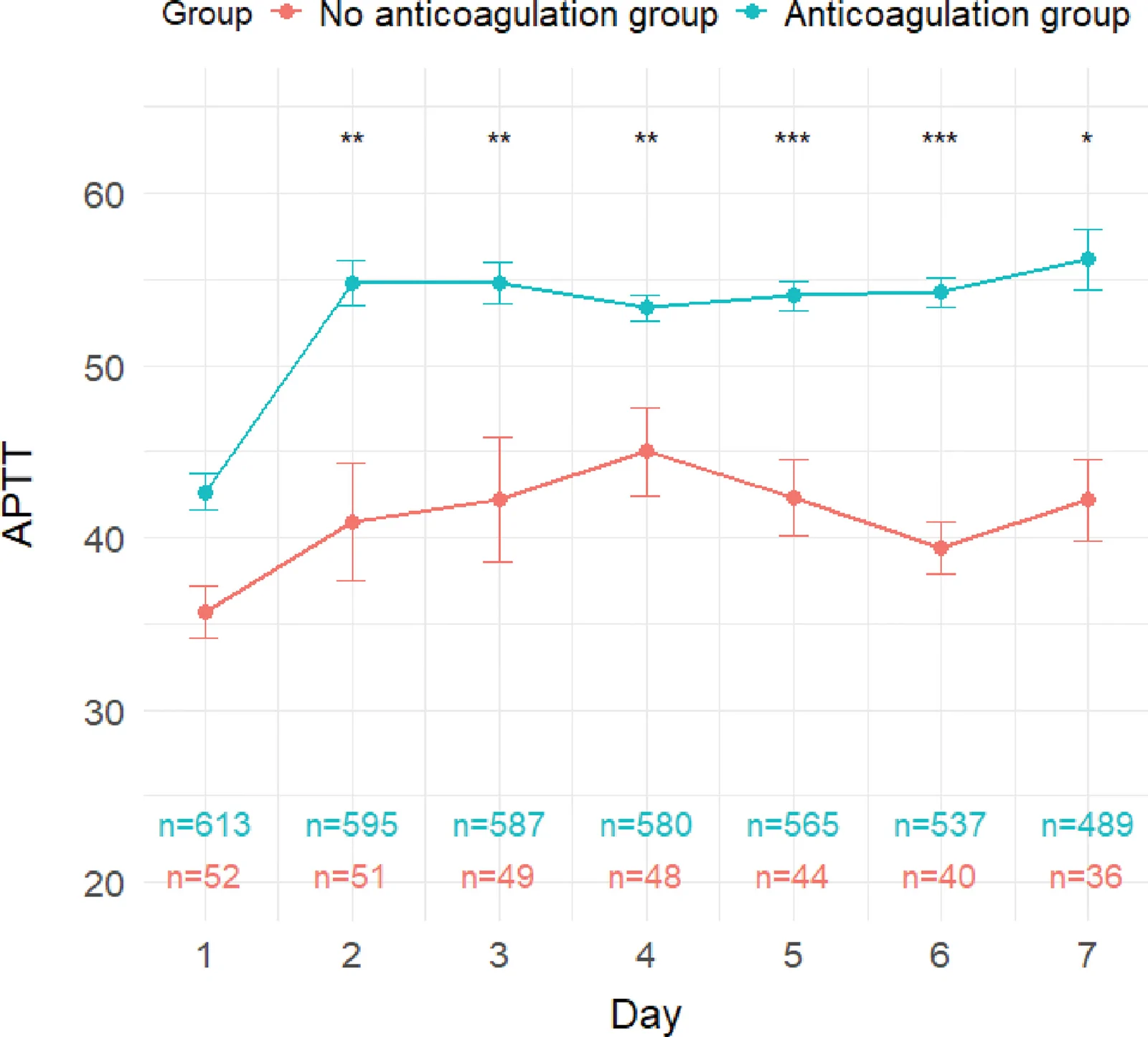
Plot of standardized mean differences before and after overlap weighting. ARDS = acute respiratory distress syndrome, APTT = activated partial thromboplastin time, SOFA = sequential organ failure assessment.

After calculating the PS, overlap weighting was applied and the balance before and after weighting was compared. The balance after weighing was generally acceptable (**Fig. 2**). The SMDs are presented in **eTable 3** (https://links.lww.com/CCM/H903). After applying overlap weighting, univariate logistic regression analysis was conducted for categorical outcomes, and the results are summarized in **Table 3**. Anticoagulation therapy was not significantly associated with 28-day survival (odds ratio [OR], 1.104; 95% CI, 0.427–2.855; *p* = 0.84), 60-day survival (OR, 0.819; 95% CI, 0.376–1.783; *p* = 0.61), or bleeding complications (OR, 0.813; 95% CI, 0.404–1.634; *p* = 0.56).

**TABLE 3.** Logistic Regression for Categorical Outcomes After Overlap Weighting

Parameters	OR	95% CI	*p*
28-d survival—anticoagulation	1.104	0.427–2.855	0.84
60-d survival—anticoagulation	0.819	0.376–1.783	0.61
Bleeding complication—anticoagulation	0.813	0.404–1.634	0.56

OR = odds ratio.

**Figure 2.**
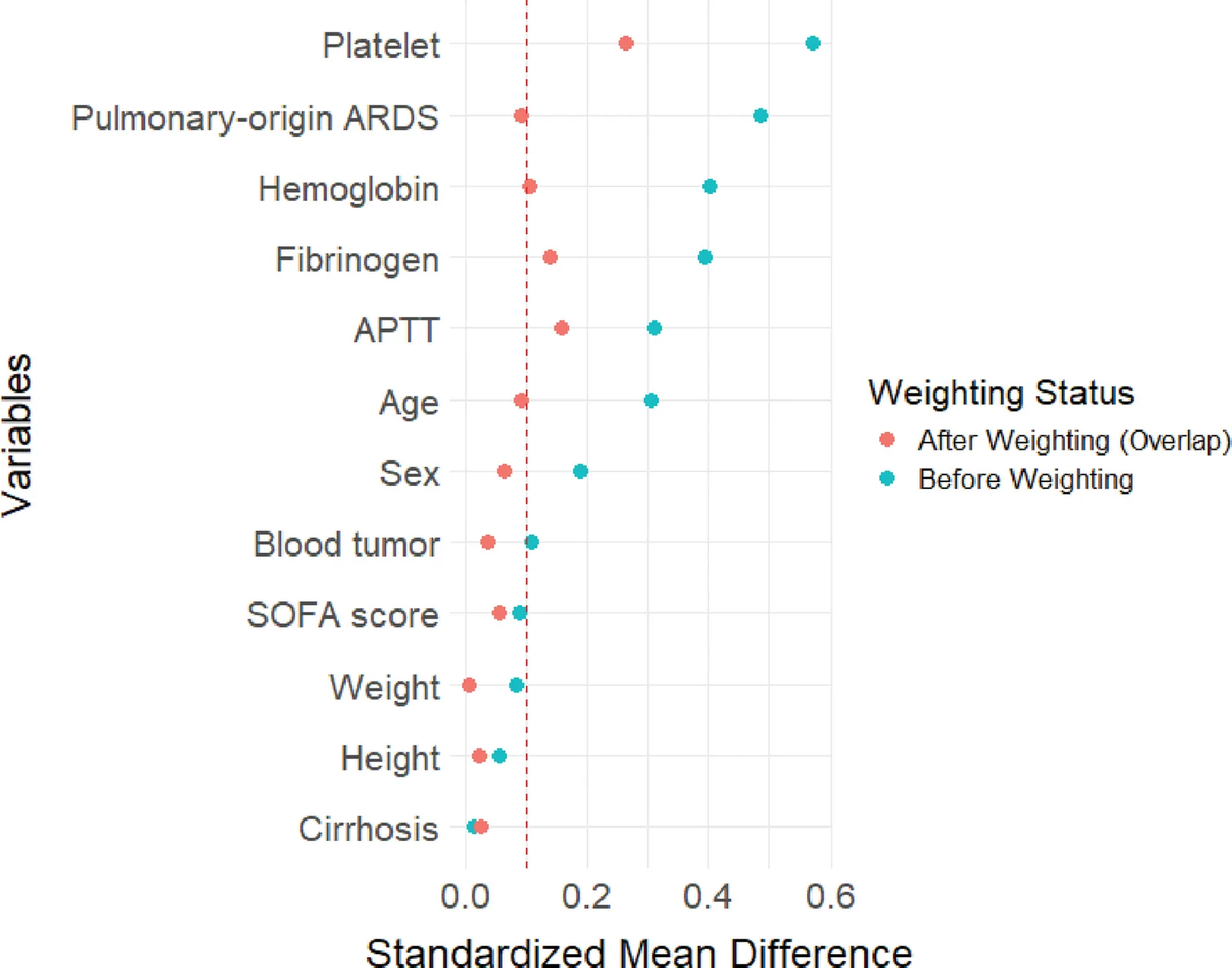
A graph showing the trends of activated partial thromboplastin time (APTT) in the two groups. Values are shown as mean ± sd. Between-group comparisons at each time point were performed using the Mann-Whitney *U* test with Benjamini–Hochberg correction for multiple comparisons. There was a significant difference in APTT between the two groups except on day 1. *p < 0.05, **p < 0.01, ***p < 0.001.

For continuous outcomes, anticoagulation was associated only with higher APTT (β 13.18, *p* < 0.01); no associations were seen for ECMO duration, circuit exchange, or transfusion volumes (**Table 4**).

**TABLE 4.** Simple Regression for Continuous Outcomes After Overlap Weighting

Parameters	β Coefficient	95% CI	*p*
ECMO duration—anticoagulation	0.142	–0.117 to 0.402	0.28
Number of ECMO circuit exchanges—anticoagulation	0.036	–0.205 to 0.276	0.77
Average activated partial thromboplastin time —anticoagulation	13.184	10.194 to 16.174	< 0.01
Red cells concentrate —anticoagulation	–0.333	–0.765 to 0.100	0.13
Fresh frozen plasma—anticoagulation	–0.382	–0.992 to 0.288	0.22
Platelet concentrate—anticoagulation	–0.345	–1.058 to 0.369	0.34

ECMO = extracorporeal membrane oxygenation.

For the sensitivity analysis, we applied the same PS. The balance after weighting was generally acceptable (**eTable 4** and **eFig. 1**, https://links.lww.com/CCM/H903). IPTW-adjusted analyses yielded results consistent with those of the main analysis (**eTables 5** and **6**, https://links.lww.com/CCM/H903).

The supplementary sensitivity analyses revealed that anticoagulant use was not associated with 28-day survival after adjusting for PS, fibrinogen level, and platelet count (**eTable 7**, https://links.lww.com/CCM/H903). Similarly, after adjusting for PS and pulmonary-origin ARDS, anticoagulant use was still not associated with 28-day survival (**eTable 8**, https://links.lww.com/CCM/H903). In the Bayesian logistic regression analysis, anticoagulant use was also found not to be associated with 28-day survival (OR 1.00; 95% CI, 0.41–2.46; **eTable 9**, https://links.lww.com/CCM/H903).

In addition, anticoagulation was not significantly associated with 28-day survival after adjustment for pump flow (OR 1.15; 95% CI, 0.31–4.19; *p* = 0.83). Pump flow was not associated with survival (OR 1.12; 95% CI, 0.49–2.56; *p* = 0.79).

Similarly, in the mixed-effects Cox model incorporating a facility-level random intercept, the association between anticoagulation strategy and 28-day mortality remained unchanged (hazard ratio 0.91; 95% CI, 0.14–3.14; *p* = 0.88), with minimal between-facility variability (random-effect variance 0.0004).

Adjustment for calendar year negligibly impacted the association between anticoagulation use and 28-day survival (OR 1.10; 95% CI, 0.29–4.24; *p* = 0.89), indicating that temporal changes in ECMO practice did not influence the findings.

Daily transfusion volumes were higher in the no-anticoagulation group on days 1 and 2 (**eFig. 2**, https://links.lww.com/CCM/H903). In terms of cumulative bleeding complication rates, the no-anticoagulation group showed periods without increase (days 2–7, 11–15, and after day 18), whereas the anticoagulation group exhibited a consistently increasing trend. Although there was a difference in bleeding events on day 1, the overall incidence across the observation period became comparable between the groups (**eFig. 3**, https://links.lww.com/CCM/H903).

## DISCUSSION

We examined the association between anticoagulation therapy and clinical outcomes in patients with ARDS receiving VV-ECMO. After adjustment using PS, there were no significant differences in the 28-day survival, 60-day survival, ECMO duration, number of ECMO circuit exchanges, bleeding complications, or transfusion requirements between the anticoagulation and no-anticoagulation groups. The sensitivity analysis yielded consistent results, further supporting the robustness of our findings.

### Strengths and Clinical Impact

To date, this study is one of the largest retrospective analyses in which the impact of anticoagulation therapy is evaluated in adult patients with ARDS supported by VV-ECMO. By leveraging a nationwide multicenter registry, we assessed real-world data from various institutions, thereby increasing the generalizability of our findings.

A key strength of our study was the use of PS weighting to mitigate confounding factors when evaluating the impact of anticoagulation therapy. The overlap weighting method ensured a well-balanced comparison between the groups, and sensitivity analysis using IPTW demonstrated consistent results, reinforcing the robustness of our findings. In addition, we examined both survival and clinically relevant secondary outcomes such as ECMO duration, circuit exchange, bleeding complications, and transfusion volume, providing a comprehensive assessment of anticoagulation use during VV-ECMO.

### Interpretation

One possible explanation for the lack of significant impact on clinical outcomes is confounding by indication, in which anticoagulation was selectively withheld in patients with a higher bleeding risk (10–13). In our cohort, patients in the no-anticoagulation group may have had a history of pre-ECMO bleeding events, which led clinicians to avoid systemic anticoagulation. In the no-anticoagulation group, the hemoglobin level, platelet count, and fibrinogen level were lower than they were in the anticoagulation group (Table 1; and eTable 1, https://links.lww.com/CCM/H903), and the incidence of bleeding complications and transfusion volume were higher on day 1 (eTables 4 and 5, https://links.lww.com/CCM/H903). Although the explicit reasons for withholding anticoagulation were not captured in the registry, these findings suggest that bleeding events may have occurred prior to or at the time of VV-ECMO initiation, potentially influencing the clinical decision to avoid anticoagulation therapy. This aligns with previous reports showing that patients with trauma, recent hemorrhage, or high-bleeding risk can sometimes be managed without systemic anticoagulation during VV-ECMO (10–13). The higher transfusion volumes in the no-anticoagulation group may reflect the need to correct pre-existing bleeding or consumptive coagulopathy at VV-ECMO initiation. These early deficits may have necessitated sustained transfusion support even after overt bleeding was controlled.

Another consideration is that thrombotic events requiring circuit exchange are relatively rare, even in the absence of systemic anticoagulation. In addition, improvements in ECMO circuit coatings and anticoagulation monitoring may have contributed to a reduced need for systemic anticoagulation (7, 27). However, the higher proportion of pulmonary-origin ARDS in the anticoagulation group may reflect a greater extent of pulmonary vascular thrombosis in this population. To address concerns that such baseline differences might confound the observed outcomes, we performed a sensitivity analysis with adjustment for ARDS origin. After adjustment, anticoagulant use was still not associated with 28-day survival (eTable 8, https://links.lww.com/CCM/H903), which suggests that the relationship between anticoagulation and survival was not confounded by ARDS etiology.

If clinical outcomes are comparable between patients who receive and those who do not receive anticoagulation therapy, one might argue that systemic anticoagulation should be universally administered to prevent rare but severe thrombotic events. However, as discussed earlier, patients in the no-anticoagulation group in our cohort may have already exhibited bleeding tendencies prior to or at the initiation of VV-ECMO. Previous studies have revealed that bleeding complications—including intracranial hemorrhage—are not uncommon in ECMO patients (2, 3) and are associated with increased mortality (28). Conversely, thrombotic complications, while serious, tend to occur less frequently (3). These findings highlight the need for a balanced and individualized anticoagulation strategy, in which the risks of bleeding and thrombosis are carefully weighed, rather than a standardized approach.

Pre-ECMO oxygenation status, reflected by arterial pH and Po_2_, was comparable between the groups. Because oxygenation saturation is a major driver of clinical decision-making in ARDS and may influence both the initiation and management of ECMO, the absence of substantial differences in pre-ECMO Po_2_ reduces the likelihood that baseline respiratory failure severity confounded the observed associations.

In our cohort, patients managed without systemic anticoagulation required substantially greater transfusion volumes and showed more pronounced coagulation abnormalities at baseline. These findings are consistent with previous case reports describing the use of no-anticoagulation VV-ECMO in patients with major bleeding risk. Therefore, it is plausible that clinicians withheld anticoagulation in patients perceived to be at high risk of bleeding, introducing confounding by indication that cannot be fully eliminated in this registry-based analysis.

In addition, anticoagulation and bleeding-management strategies vary among the 24 participating centers, and this institutional heterogeneity may have influenced decisions regarding whether to administer systemic anticoagulation. These practice patterns are partially reflected in proxy variables such as platelet count, fibrinogen level, APTT, and transfusion requirements, all of which were imbalanced to some extent despite overlap weighting. However, a sensitivity analysis using a random-effects model with facility_code as a random intercept yielded results consistent with the main analysis, suggesting that between-site variability was unlikely to meaningfully alter the observed association.

In addition, adjustment for calendar year did not alter the association between anticoagulation use and 28-day survival. This suggests that temporal variation in VV-ECMO practice did not materially influence our findings.

### Limitations

Our study has certain limitations. First, as a retrospective observational study, there is a possibility of residual confounding, despite the use of PS-based overlap weighting. Confounding by indication may have influenced the results as patients in the no-anticoagulation group were likely to have a higher risk of bleeding, leading clinicians to withhold anticoagulation.

Second, there was not standardization of anticoagulation strategies across centers.

Third, in our study, anticoagulation was treated as a binary variable (yes/no) without accounting for different anticoagulation regimens.

Finally, we did not assess long-term outcomes, such as functional status or ECMO-related thrombosis after decannulation.

## CONCLUSIONS

There is no link between systemic anticoagulation and short-term survival. These findings support the feasibility of anticoagulation-free VV-ECMO in selected high-bleeding-risk patients, but do not establish safety or equivalence. Future studies should determine optimal anticoagulation strategies.

## ACKNOWLEDGMENTS

Japan Chest CT for acute respiratory distress syndrome requiring venovenous extracorporeal membrane oxygenation (J-CARVE) registry group authors are as follows: Mitsuaki Nishikimi (Department of Emergency and Critical Care Medicine, Graduate School of Biomedical and Health Sciences, Hiroshima University, Hiroshima, Japan); Shinichiro Ohshimo (Department of Emergency and Critical Care Medicine, Graduate School of Biomedical and Health Sciences, Hiroshima University, Hiroshima, Japan); Jun Hamaguchi (Department of Critical Care and Emergency Medicine, Tokyo Metropolitan Tama Medical Center, Tokyo, Japan); Kazuki Matsumura (Department of Critical Care and Emergency Medicine, Tokyo Metropolitan Tama Medical Center, Tokyo, Japan); Kenji Fujizuka (Advanced Medical Emergency Department and Critical Care Center, Japan Red Cross Maebashi Hospital, Maebashi, Japan); Yoshihiro Hagiwara (Department of Emergency Medicine and Critical Care Medicine, SAISEIKAI Utsunomiya Hospital, Utsunomiya, Japan); Ryuichi Nakayama (Department of Emergency Medicine, Sapporo Medical University, Sapporo, Japan); Naofumi Bunya (Department of Emergency Medicine, Sapporo Medical University, Sapporo, Japan); Junichi Maruyama (Department of Emergency medicine and Critical care, Fukuoka University Hospital, Fukuoka, Japan); Takayuki Ogura (Department of Emergency Medicine and Critical Care Medicine, SAISEIKAI Utsunomiya Hospital, Utsunomiya, Japan); Mitsunobu Nakamura (Advanced Medical Emergency Department and Critical Care Center, Japan Red Cross Maebashi Hospital, Maebashi, Japan); Keiki Shimizu (Department of Critical Care and Emergency Medicine, Tokyo Metropolitan Tama Medical Center, Tokyo, Japan); Mamoru Masuda (Advanced Medical Emergency Department and Critical Care Center, Japan Red Cross Maebashi Hospital, Maebashi, Japan); Junki Ishii (Department of Emergency and Critical Care Medicine, Graduate School of Biomedical and Health Sciences, Hiroshima University, Hiroshima, Japan); and Nobuaki Shime (Department of Emergency and Critical Care Medicine, Graduate School of Biomedical and Health Sciences, Hiroshima University, Hiroshima, Japan).

## Supplementary Material
