# Supplementary material for: Association Between Systemic Anticoagulation and Outcomes in Patients With Acute Respiratory Distress Syndrome Receiving Venovenous Extracorporeal Membrane Oxygenation: Insights From a Multicenter Propensity-Weighted Study
Source: Crit Care Med. 2026 Feb 19;54(5):1103–13. doi: 10.1097/CCM.0000000000007071 (PMC13750810; doi:10.1097/CCM.0000000000007071)
Supplement: Supplementary file 1 [file ccm-54-1103-s001.pdf]

Supplementary file

eTable 1. Baseline patient characteristics

Parameters	Anticoagulation group (N = 641)	No anticoagulation group (N = 54)	P value
Age, years	60.0 (50.0–68.0)	58.5 (47.3–66.0)	0.17
Male, n (%)	489 (76.3)	44 (81.5)	0.48
Height, cm	167.0 (160.0–172.0)	165.0 (160.0–170.0)	0.40
Weight, kg	72.2 (61.0–85.1)	69.0 (58.0–80.0)	0.17
Past medical history			
Hypertension, n (%)	254 (39.6)	13 (24.1)	0.03
Diabetes, n (%)	205 (32.0)	9 (16.7)	0.03
COPD, n (%)	43 (6.7)	0 (0)	0.04
Asthma, n (%)	46 (7.2)	2 (3.7)	0.57
Interstitial lung disease, n (%)	26 (4.1)	1 (1.9)	0.71
Chronic heart failure, n (%)	37 (5.8)	0 (0)	0.10
Chronic kidney disease, n (%)	55 (8.6)	3 (5.6)	0.61
Blood tumor, n (%)	14 (2.2)	2 (3.7)	0.36
Cirrhosis, n (%)	11 (1.7)	1 (1.9)	1.00
Dementia, n (%)	3 (0.5)	0 (0)	1.00
Collagen disease, n (%)	37 (5.8)	1 (1.9)	0.35
Pulmonary-origin ARDS, n (%)	605 (94.4)	44 (81.5)	<0.01
Laboratory data before ECMO			
pH	7.3 (7.2–7.4)	7.3 (7.2–7.4)	0.46
Lactate, mmol/L	1.7 (1.2–2.4)	2.0 (1.3–4.4)	0.02
White blood cell count, $\times 10^3/\mu L$	11.3 (7.7–16.5)	11.9 (6.5–16.2)	0.75

Hemoglobin, g/dL	12.5 (10.5–14.1)	11.0 (9.7–13.0)	<0.01
Platelet count, $\times 10^3/\mu L$	184.0 (115.0–257.0)	116.0 (75.0–163.0)	<0.01
C-reactive protein, mg/dL	12.6 (4.9–21.7)	8.2 (2.1–16.5)	0.05
T-bil, mg/dL	0.7 (0.5–1.1)	0.8 (0.5–2.2)	0.02
Albumin, mg/dL	2.5 (2.0–2.8)	2.5 (2.3–3.0)	0.07
Creatinine, mg/dL	0.9 (0.7–1.5)	1.0 (0.7–1.9)	0.43
APTT, s	35.0 (29.0–46.5)	32.0 (28.9–37.3)	0.09
FDP, μg/mL (N = 398, 36)	14.1 (6.6–36.1)	24.4 (8.9–50.2)	0.30
Fibrinogen, mg/dL (N = 588, 49)	509.0 (347.8–657.5)	365.0 (254.0–614.0)	0.01
BNP, pg/mL (N = 171, 19)	66.1 (27.3–193.7)	110.0 (45.6–175.4)	0.43
SOFA score	10.0 (7.0–12.0)	10.5 (8.0–13.0)	0.24
Duration between MV and ECMO initiation, days	2.0 (1.0–5.0)	2.0 (1.0–5.0)	0.59
Gas exchange status before ECMO			
FiO_2	1.0 (0.8–1.0)	1.0 (1.0–1.0)	0.04
pO_2, torr	71.4 (60.0–86.1)	72.0 (56.8–83.0)	0.24
P/F ratio	81.5（64.0–104.1)	81.4 (62.0–97.0)	0.44
pCO_2, torr	49.0 (41.0–62.7)	47.0 (37.1–62.7)	0.52
MV setting before ECMO			
PEEP, cmH_2O	12.0 (10.0–15.0)	10.0 (8.0–15.0)	0.02
Tidal volume, mL (N = 496, 46)	400.0 (322.5–477.3)	380.0 (301.5–450.0)	0.21

COPD, chronic obstructive pulmonary disease; ECMO, extracorporeal membrane oxygenation; ARDS, acute respiratory distress syn drome; T-bil, total bilirubin; APTT, activated partial thromboplastin time; FDP, fibrinogen/fibrin degradation products; BNP, brain natriuretic peptide; SOFA, sequential organ failure assessment; MV, mechanical ventilation; PEEP, positive end-expiratory pressure

eTable 2. ECMO settings at initiation

Parameters	Anticoagulation group (N = 641)	No anticoagulation group (N = 54)	P value
ECMO FiO_2, %	1.0 (1.0–1.0)	1.0 (1.0–1.0)	0.24
Rotation speed, rpm	2660.0 (2078.0–3000.0)	2802.5 (2332.5–3095.0)	0.20
Pump flow rate, L/min	4.0 (3.5–4.2)	4.1 (3.5–4.7)	< 0.01
Gas flow rate, L/min	3.5 (2.5–4.0)	3.2 (3.0–4.0)	0.67

ECMO, extracorporeal membrane oxygenation

eTable 3. Standardized mean differences before and after overlap weighting

Parameters	Before weighting	After weighting
Age	0.31	0.09
Sex	0.19	0.06
Height	0.06	0.02
Weight	0.08	<0.01
Blood tumor	0.11	0.04
Cirrhosis	0.01	0.02
Pulmonary-origin ARDS	0.49	0.09
APTT	0.31	0.16
Hemoglobin	0.40	0.11
Platelets	0.57	0.26
Fibrinogen	0.39	0.14
SOFA score	0.09	0.06

ARDS, acute respiratory distress syn drome; APTT, activated partial thromboplastin time; SOFA, sequential organ failure assessment

eTable 4. Standardized mean differences before and after inverse probability of treatment weighting

Parameters	Before weighting	After weighting
Age	0.31	0.02
Sex	0.19	0.17
Height	0.06	0.02
Weight	0.08	0.09
Blood tumor	0.11	0.04
Cirrhosis	0.01	0.01
Pulmonary-origin ARDS	0.49	0.02
APTT	0.31	0.01
Hemoglobin	0.40	0.26
Platelets	0.57	0.77
Fibrinogen	0.39	0.34
SOFA score	0.09	0.12

ARDS, acute respiratory distress syn drome; APTT, activated partial thromboplastin time; SOFA, sequential organ failure assessment

eFigure 1. Standardized mean differences before and after inverse probability of treatment weighting

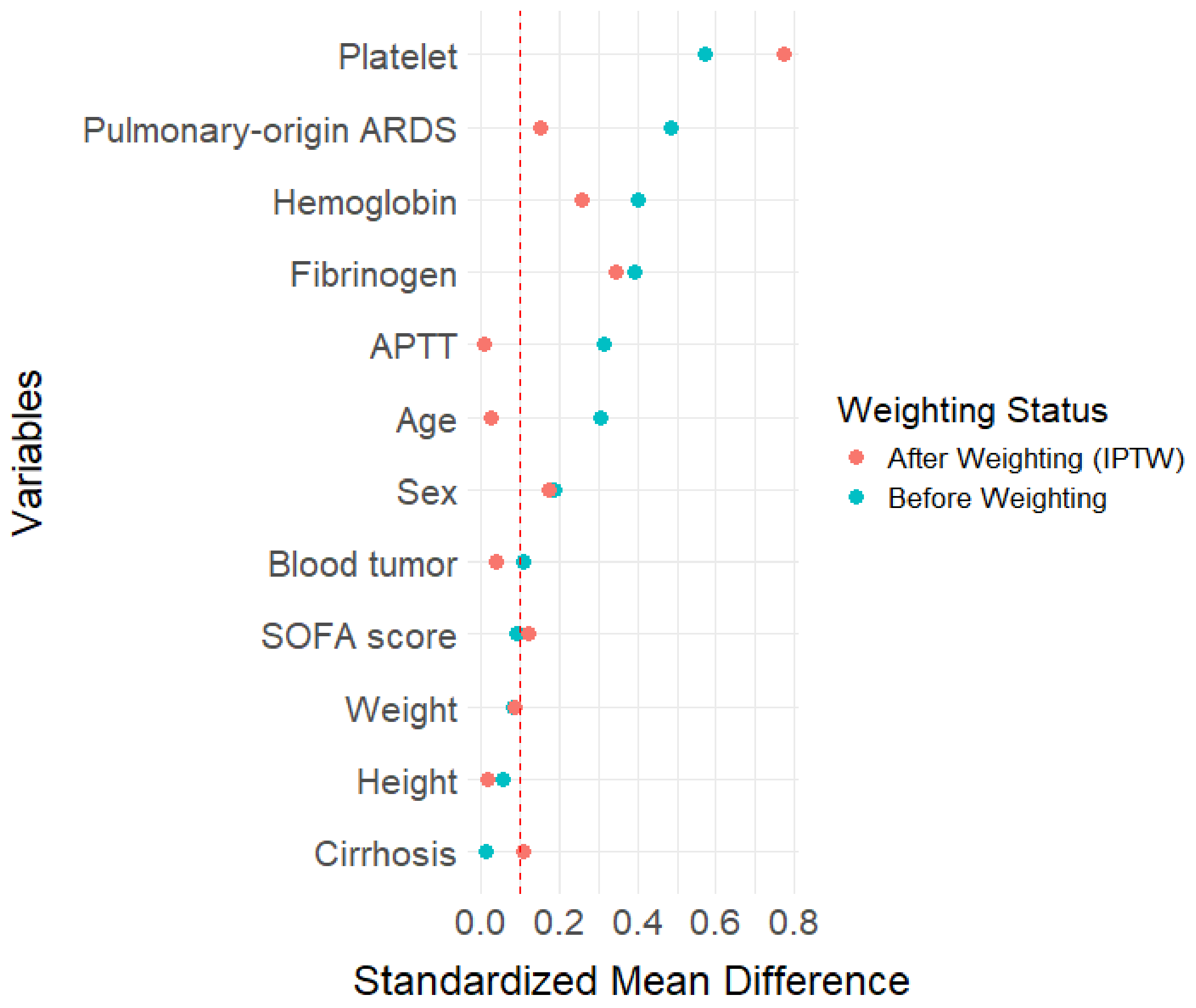

Plot of standardized mean differences before and after inverse probability of treatment weighting. ARDS, acute respiratory distress syndrome; APTT, activated partial thromboplastin time; SOFA, sequential organ failure assessment

eTable 5. Logistic regression for categorical outcomes after inverse probability of treatment weighting

**Parameters**	**Odds ratio**	**95% CI**	**P value**
28-day survival—Anticoagulation	1.748	0.614–4.974	0.30
60-day survival—Anticoagulation	0.879	0.353–2.188	0.78
Bleeding complication—Anticoagulation	0.756	0.340–1.677	0.49

CI, confidence interval

eTable 6. Simple regression for continuous outcomes after inverse probability of treatment weighting

Parameters	β coefficient	95% CI	P value
ECMO duration—Anticoagulation	0.104	–0.150 to 0.359	0.42
Number of ECMO circuit exchanges—Anticoagulation	–0.115	–0.331 to 0.100	0.29
Average APTT—Anticoagulation	12.735	9.428 to 16.041	<0.01
RCC—Anticoagulation	–0.358	–0.863 to 0.147	0.16
FFP—Anticoagulation	–0.343	–0.950 to 0.265	0.27
PLT—Anticoagulation	–0.351	–1.060 to 0.358	0.33

CI, confidence interval; ECMO, extracorporeal membrane oxygenation; APTT, activated partial thromboplastin time; RCC, red cells concentrate; FFP, fresh frozen plasma; PLT, platelet concentrate

eTable 7. Logistic regression with adjustment for fibrinogen level and platelet count

Parameters	Odds ratio	95% CI	P value
Propensity score	0.129	0.004 to 2.722	0.21
Anticoagulation	1.099	0.418 to 2.553	0.84
Fibrinogen	1.001	1.000 to 1.002	0.29
Platelets	1.003	1.000 to 1.006	0.02

CI, confidence interval

eTable 8. Logistic regression with adjustment for pulmonary-origin ARDS

Parameters	Odds ratio	95% CI	P value
Propensity score	0.423	0.015–8.825	0.59
Anticoagulation	1.096	0.416–2.545	0.84
Pulmonary-origin ARDS	1.623	0.552–4.297	0.35

ARDS, acute respiratory distress syndrome; CI, confidence interval

eTable 9. Bayesian logistic regression analysis for 28-day survival

**Parameters**	**Odds ratio (95% CI)**	$\hat{R}$	**Bulk ESS**	**Tail ESS**
Propensity score	0.82 (0.07–9.02)	1.00	2,696	2,453
Anticoagulation	1.00 (0.41–2.46)	1.00	3,314	2,714

CI, confidence interval; ESS, effective sample size

eFigure 2. Mean daily red cell concentrate transfusion volume by group

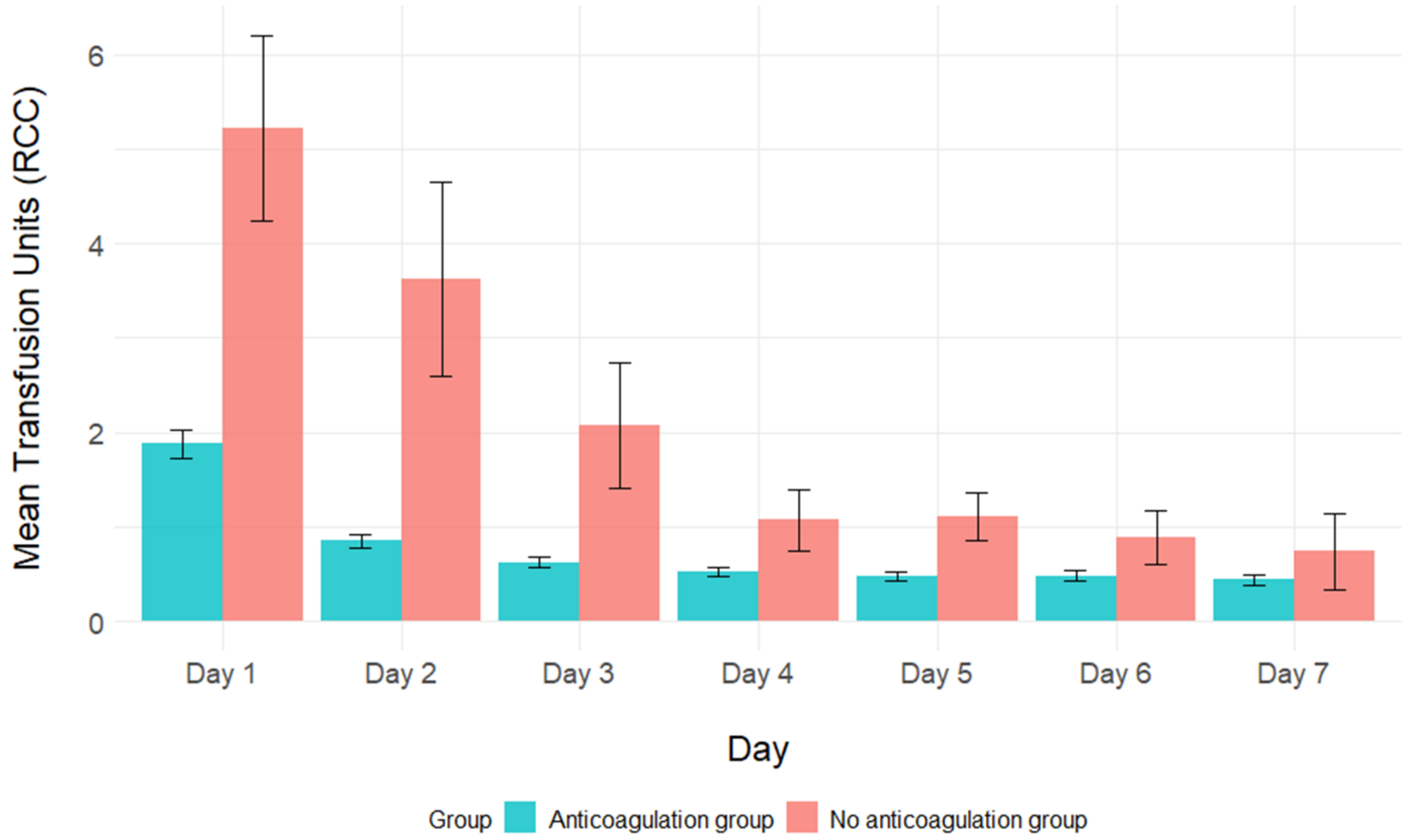

The figure illustrates daily red cell concentrate transfusion volumes administered to patients receiving VV-ECMO, stratified by anticoagulant use. RCC, red cell concentrate

eFigure 3. Daily cumulative bleeding rate by group

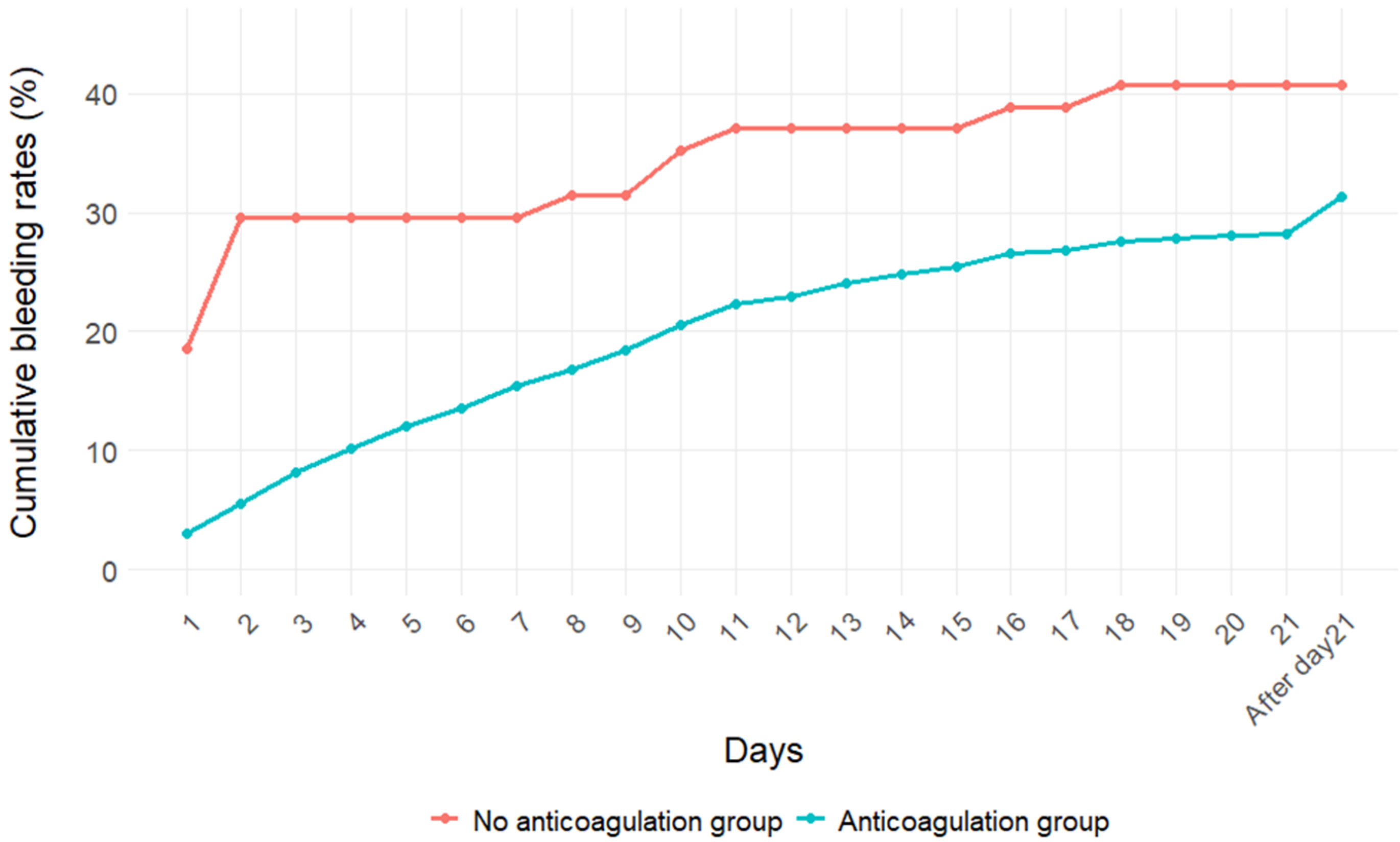

The figure shows the cumulative incidence of hemorrhagic complications over time in the anticoagulation and no anticoagulation groups.
